# Vasopressin in septic shock: effects on pancreatic, renal, and hepatic blood flow

**DOI:** 10.1186/cc6197

**Published:** 2007-12-13

**Authors:** Vladimir Krejci, Luzius B Hiltebrand, Stephan M Jakob, Jukka Takala, Gisli H Sigurdsson

**Affiliations:** 1 Department of Anesthesiology, Washington University School of Medicine, Campus Box 8054, St. Louis, MO 63110, USA; 2 Department of Anesthesiology, University of Bern, Inselspital, CH-3010 Bern, Switzerland; 3 Department of Intensive Care Medicine, University of Bern, Inselspital, CH-3010 Bern, Switzerland; 4 Department of Anesthesia & Intensive Care Medicine, Landspitali University Hospital, Hringbraut, IS 101 Reykjavik, Iceland, and University of Iceland, Reykjavik, Iceland

## Abstract

**Introduction:**

Vasopressin has been shown to increase blood pressure in catecholamine-resistant septic shock. The aim of this study was to measure the effects of low-dose vasopressin on regional (hepato-splanchnic and renal) and microcirculatory (liver, pancreas, and kidney) blood flow in septic shock.

**Methods:**

Thirty-two pigs were anesthetized, mechanically ventilated, and randomly assigned to one of four groups (*n *= 8 in each). Group S (sepsis) and group SV (sepsis/vasopressin) were exposed to fecal peritonitis. Group C and group V were non-septic controls. After 240 minutes, both septic groups were resuscitated with intravenous fluids. After 300 minutes, groups V and SV received intravenous vasopressin 0.06 IU/kg per hour. Regional blood flow was measured in the hepatic and renal arteries, the portal vein, and the celiac trunk by means of ultrasonic transit time flowmetry. Microcirculatory blood flow was measured in the liver, kidney, and pancreas by means of laser Doppler flowmetry.

**Results:**

In septic shock, vasopressin markedly decreased blood flow in the portal vein, by 58% after 1 hour and by 45% after 3 hours (*p *< 0.01), whereas flow remained virtually unchanged in the hepatic artery and increased in the celiac trunk. Microcirculatory blood flow decreased in the pancreas by 45% (*p *< 0.01) and in the kidney by 16% (*p *< 0.01) but remained unchanged in the liver.

**Conclusion:**

Vasopressin caused marked redistribution of splanchnic regional and microcirculatory blood flow, including a significant decrease in portal, pancreatic, and renal blood flows, whereas hepatic artery flow remained virtually unchanged. This study also showed that increased urine output does not necessarily reflect increased renal blood flow.

## Introduction

Low-dose vasopressin has been proposed for treatment of severe hypotension in septic shock that is otherwise unresponsive to high doses of alpha-adrenergic agents [1,2]. To date, smaller controlled studies of human subjects receiving low-dose vasopressin in septic shock have been rather encouraging, but adverse events, possibly related to the use of vasopressin, have also been reported [3,4].

Vasopressin can produce intense vasoconstriction that is independent of tissue oxygenation and metabolism [5]. The capacity of vasopressin to decrease mesenteric and portal blood flow has been demonstrated by its efficacy in reducing gastrointestinal bleeding [6], including hemorrhage from blunt liver trauma [7,8]. The effects of vasopressin were well documented in the 1970s and 1980s in human [9] and animal [10-12] studies, but this was mostly in non-septic conditions and with doses significantly exceeding what today is considered to be a 'safe' range.

Recently published results from animal studies have confirmed previous findings that high doses of vasopressin (greater than 0.1 units per minute) clearly redistribute regional blood flows and decrease tissue oxygenation [13,14]. However, reported effects of low-dose vasopressin on regional blood flow and metabolism are more conflicting and range from 'deleterious' [15] to increased mesenteric blood flow and beneficial effects on tissue metabolism [16].

The effects of low-dose vasopressin on other organs, such as the pancreas, are largely unknown. Decreased blood flow in the pancreas was found when high doses of vasopressin were infused under non-septic conditions [12], but the effects of low-dose vasopressin on the pancreas in septic shock have not been studied. The pancreas appears to be particularly vulnerable to low flow as a result of cardiogenic shock [17], hypovolemia [18,19], and sepsis [20]. Prolonged pancreatic ischemia secondary to hypovolemia may cause secretory dysfunction, edema, and inflammation [18].

Vasopressin has been reported to increase urine output [21,22] and creatinine clearance [23] in septic subjects. Low-dose vasopressin did not decrease total renal blood flow in endotoxemic pigs. However, it has been found to cause redistribution of intrarenal blood flow, resulting in a reduction of medullary blood flow [24,25] even with physiologic plasma levels.

We hypothesized that increasing systemic blood pressure by administering vasopressin in fluid-resuscitated experimental septic shock would result in a substantial redistribution of regional blood flow within the splanchnic region and, consequently, in altered microcirculatory blood flow in abdominal organs. Thus, the aim of this study was to compare changes in systemic blood flow with changes in regional splanchnic blood flow and microcirculatory blood flow in the liver, kidney, and pancreas during administration of low-dose vasopressin in fluid-resuscitated septic shock in pigs.

## Materials and methods

This study was performed according to the National Institutes of Health (Bethesda, MD, USA) guidelines for the care and use of experimental animals. The protocol was approved by the animal ethics committee of Canton Bern, Switzerland.

Thirty-two domestic pigs (weight, 28 to 32 kg) were fasted overnight but were allowed free access to water. The pigs were sedated with intramuscular ketamine (20 mg/kg) and xylazinum (2 mg/kg). After induction of anesthesia with intravenous metomidate (5 mg/kg) and azaperan (2 mg/kg), the pigs were orally intubated and ventilated with oxygen in air (fraction of inspired oxygen [FiO_2_] = 0.40). Inhaled concentration of oxygen was continuously monitored with a multi-gas analyzer (S/5™ Critical Care Monitor; Datex-Ohmeda, part of GE Healthcare, Little Chalfont, Buckinghamshire, UK). Anesthesia was maintained with continuous intravenous infusions of midazolam (0.5 mg/kg per hour), fentanyl (20 μg/kg per hour), and pancuronium (0.3 mg/kg per hour) to simulate clinical conditions as closely as possible. The animals were ventilated with a volume-controlled ventilator with a positive end-expiratory pressure (PEEP) of 5 cm H_2_O (Servo 900C; Siemens, Dietikon, Switzerland). Tidal volume was kept at 10 to 15 mL/kg and the respiratory rate was adjusted (14 to 16 breaths per minute) to maintain end-tidal carbon dioxide tension (arterial carbon dioxide partial pressure, PaCO_2_) at 40 ± 4 mm Hg. The stomach was emptied with an orogastric tube.

### Surgical preparation

Indwelling catheters were inserted through a left cervical cut-down into the thoracic aorta and vena cava superior. A balloon-tipped catheter was inserted into the pulmonary artery through the right external jugular vein. Location of the catheter tip was determined by observing the characteristic pressure trace on the monitor as it was advanced through the right heart into the pulmonary artery.

With the pig in the supine position, a midline laparotomy was performed. A catheter was inserted into the urinary bladder for drainage of urine. A second catheter was inserted into the mesenteric vein for blood sampling. The superior mesenteric artery, the celiac trunk, and the left renal artery were identified close to their origin at the aorta.

After the vessels were dissected free of the surrounding tissues, pre-calibrated ultrasonic transit time flow probes (Transonic Systems Inc., Ithaca, NY, USA) were placed around the vessels and connected to an ultrasound blood flow meter (T 207; Transonic Systems Inc.). Additional ultrasonic transit time probes were placed around the portal vein and the hepatic artery. Small custom-made laser Doppler flow probes (Oxford Optronix Ltd, Oxford, UK) were attached to the liver capsule and the surface of the left kidney. A third laser Doppler flow probe was attached to the pancreas. Six additional laser Doppler flow probes were sutured to the mucosa and serosa of the stomach, jejunum, and colon, and the data from these were presented elsewhere [26].

Twenty grams of autologous feces was collected from the colon and used later to induce peritonitis and septic shock in selected animals (the two septic groups). The colon incision was then closed with continuous sutures. The laser Doppler flowmetry (LDF) probes on the liver and the kidney were attached to the surface of each organ with six blunt needles per probe. The LDF probe on the pancreas was attached with six microsutures. The signal from the laser Doppler flow meter was visualized on a computer monitor. Care was taken to ensure continuous and steady contact with the tissue under investigation, preventing motion disturbance from respiration and gastrointestinal movements throughout the experiment. Once the experiment was started, care was taken to avoid any movement of the LDF probes and to avoid any pressure, traction, or injury to the tissue under investigation during the experiment. At the end of the surgical preparation, two large-bore tubes (32 French) were placed with the tip in the abdominal cavity before the laparotomy was closed.

During surgery, the animals received lactated Ringer's solution 15 to 20 mL/kg per hour, which kept central venous and pulmonary capillary wedge pressures (PCWPs) constant between 6 and 8 mm Hg. Body temperature was maintained at 37.5°C ± 0.5°C by the use of a warming mattress and a patient air warming system (Warm Touch 5700; Mallinckrodt, Hennef, Germany). After the surgical preparation was completed, the animals were allowed to stabilize for 45 to 60 minutes.

### Experimental design

This study was planned using a factorial design. The animals were randomly assigned into one of the following groups:

#### Group C

Non-septic control group (*n *= 8): After baseline measurements, lactated Ringer's solution was given at a rate of 20 mL/kg per hour throughout the experiment.

#### Group V

Non-septic vasopressin control group (*n *= 8): After baseline measurements, the animals were treated the same way as animals in group C, except at 300 minutes a continuous intravenous infusion of ornithin-8 vasopressin (POR-8^®^; Ferring, Wallisellen, Switzerland) was started at a rate of 0.06 IU/kg per hour and maintained for another 180 minutes.

#### Group S

Septic control group (*n *= 8): After baseline measurements, the animals were exposed to fecal peritonitis by instillation of 20 g of autologous feces suspended in 200 mL of warm (37°C) 5% dextrose through the abdominal tubes. Simultaneously, administration of lactated Ringer's solution was discontinued. After 240 minutes of peritonitis and development of septic shock, an intravenous fluid bolus (4% gelatine; Physiogel^® ^molecular weight 30,000; B. Braun Medical, Sempach, Switzerland) of 15 mL/kg was given over the span of 45 minutes, followed by intravenous lactated Ringer's solution at a rate of 20 mL/kg per hour until the end of the study.

#### Group SV

Septic test group treated with vasopressin (*n *= 8): The animals were treated in the same way as the septic control group (group C), except that at 300 minutes a continuous intravenous infusion of ornithin-8-vasopressin was started at a rate of 0.06 IU/kg per hour and maintained for another 180 minutes. Four hundred eighty minutes after baseline measurement, all animals were sacrificed with an intravenous injection of 20 mmol KCl.

### Hemodynamic monitoring

Mean arterial blood pressure (MAP) (mm Hg), central venous pressure (CVP) (mm Hg), mean pulmonary artery pressure (PAP) (mm Hg), and PCWP (mm Hg) were recorded with quartz pressure transducers. Heart rate (HR) was measured from the electrocardiogram. HR, MAP, PAP, and CVP were displayed continuously on a multi-modular monitor (S/5™, Critical Care Monitor; Datex-Ohmeda). Cardiac output (CO) (liters per minute) was updated every 60 seconds using a thermodilution method. The value was displayed continuously on a continuous CO monitor (Vigilance CCO Monitor; Edwards Lifesciences, S.A., Horw, Switzerland).

### Respiratory monitoring

Expired minute volume, tidal volume, respiratory rate, peak and end inspiratory pressures, PEEP (cm H_2_O), inspired and end-tidal carbon dioxide concentrations (mm Hg), and inspired (FiO_2_) and expired oxygen fractions were monitored continuously throughout the study.

### Laser Doppler flowmetry

LDF is an established non-invasive technique for continuous monitoring of the microcirculation *in vivo *and has been shown not to interfere with blood flow in the tissue under investigation [20,27]. The LDF data were acquired online with a sampling rate of 10 Hz via a multichannel interface (Mac Paq MP 100; Biopac Systems, Inc., Goleta, CA, USA) with acquisition software (Acqknowledge 3.2.1.; Biopac Systems, Inc.) installed in a portable computer.

Laser Doppler flow meters are not calibrated to measure absolute blood flow; rather, they indicate microcirculatory blood flow in arbitrary perfusion units. Due to relatively large variability in baseline values, the results are usually expressed as changes relative to baseline [28], which was also the case in this study. The quality of the LDF signal was controlled online by visualization on a computer screen, so that motion artifacts and noise due to inadequate probe attachment could be immediately detected and corrected before the measurements started.

### Ultrasonic transit time flowmetry

Blood flow in the hepatic artery, renal artery, celiac trunk, and portal vein was continuously measured in all animals throughout the experiments by means of ultrasonic transit time flowmetry (mL per minute) and an HT 206 flow meter (Transonic Systems Inc.).

### Laboratory analysis

For all animals, arterial, mixed venous, and mesenteric venous blood samples were withdrawn at each measurement point from the indwelling catheters and immediately analyzed in a blood gas analyzer (ABL 620; Radiometer A/S, Brønshøj, Denmark) for partial pressure of oxygen (mm Hg), partial pressure of carbon dioxide (mm Hg), pH, lactate (mmol/L), oxygen saturation of hemoglobin (%), base excess (mmol/L), and total hemoglobin concentration (g/L). All values were adjusted to body temperature.

### Data analysis and calculations

Cardiac index (CI), systemic vascular resistance (SVR), and flows in the celiac trunk, portal vein, and hepatic and renal arteries were indexed to body weight. SVR index was calculated as: SVR index = (MAP - CVP)/CI [13,15].

Systemic oxygen delivery index (DO_2_I sys) as well as the derived splanchnic oxygen delivery indices (portal venous [DO_2_I PV], hepatic arterial [DO_2_I HA], total [DO_2_I liver], and renal arterial [DO_2_I kidney] oxygen delivery indices) were calculated: DO_2_I = (indexed flow) × CaO_2_, where CaO_2 _is the arterial oxygen content: CaO_2 _= (PaO_2 _× 0.003) + (Hb × SaO_2 _× 1.36). PaO_2 _is arterial oxygen partial pressure, Hb is the hemoglobin concentration, and SaO_2 _is the arterial oxygen saturation. Systemic (total body) oxygen consumption index was calculated as follows: VO_2_I = CI × (CaO_2 _- CvO_2_), where CvO_2 _is the mixed venous oxygen content.

### Statistical analysis

The data are presented as mean ± standard deviation for the four study groups. Differences between the four groups were assessed by analysis of variance (ANOVA) for repeated measurements using one dependent variable, one grouping factor (controls, controls with vasopressin, sepsis, and sepsis with vasopressin), and one within-subject factor (time). When there was a significant group-time interaction, the effect of vasopressin was assessed separately in the two groups with and without sepsis by again using ANOVA for repeated measurements. In this design, a significant time-group interaction is interpreted as an effect of vasopressin. Finally, the effects of vasopressin in the groups with and without sepsis were compared by calculating the area under the variable-time curve during vasopressin infusion (Mann-Whitney test). Calculations for microcirculatory blood flow were performed using changes relative to baseline (t = 0 minutes). Absolute values were used for all other calculations. All the *p *values given in the Results section represent the calculated *p *value for the time-group interaction, unless otherwise stated.

## Results

**Figure 1:**
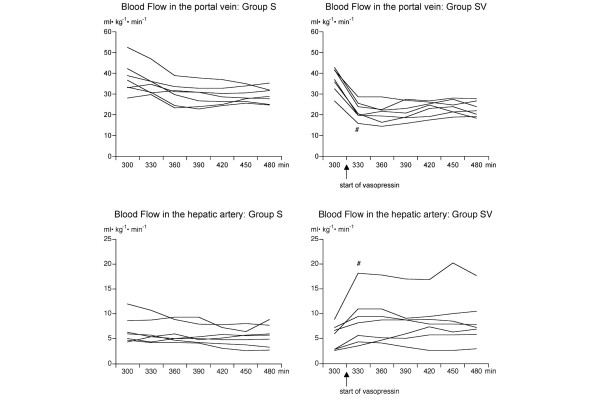
Blood flow in the portal vein and in the hepatic artery measured with ultrasonic transit time flowmetry during septic shock. A continuous infusion of vasopressin (0.06 IU/kg per hour) was started at t = 300 minutes in animals in group SV. Animals in group S received intravenous saline only. Results are presented as individual curves. Portal venous and hepatic arterial blood flows are indexed to body weight. There was a significantly greater decrease in portal venous blood flow in group SV than in group S (*p *< 0.01). Hepatic artery blood flow remained virtually unchanged in all animals in group S and in five out of eight in group SV. Three animals in group SV may have had some hepatic arterial buffer response. ^#^*p *< 0.01 compared with t = 300 minutes. Group S, septic control group; group SV, septic test group treated with vasopressin.

**Figure 2:**
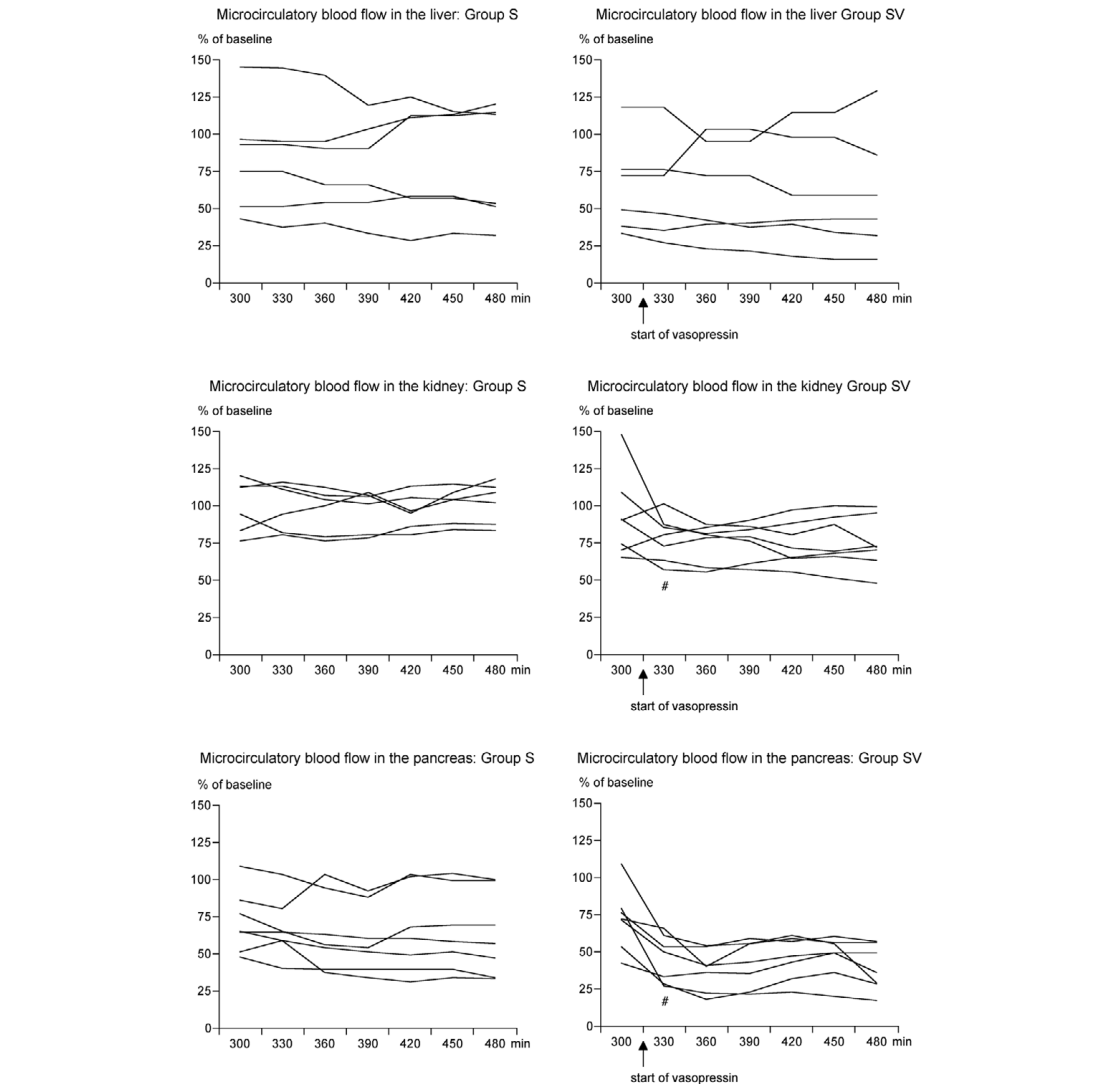
Microcirculatory blood flow of the liver, the pancreas, and the kidney measured with laser Doppler flowmetry during septic shock. A continuous infusion of vasopressin (0.06 IU/kg per hour) was started at t = 300 minutes in animals in group SV. Animals in group S received intravenous saline only. Results are presented as individual curves. Microcirculatory blood flow is expressed as changes relative to the baseline values (t = 0 minutes). #*p *< 0.01 compared to t = 300 minutes. Group S, septic control group; group SV, septic test group treated with vasopressin.

Systemic, regional, and local parameters recorded during the development of septic shock and during fluid resuscitation but before t = 300 minutes are presented in Appendix 1. Data recorded after t = 300 minutes until end of the study at t = 480 minutes are presented below and in Tables 1, 2, 3 and Figures 1 and 2. Three series of LDF measurements from the liver (one each in groups V, S, and SV) and two series from the kidney (one from group C and another from group S) had to be excluded because of excessive motion artifacts and loss of optical coupling to the tissue.

**Table 1:** Systemic hemodynamics and metabolic variables during infusion of vasopressin

Time		300 minutes	360 minutes	480 minutes
Heart rate (beats per minute)^a,b^				
	Group C	126 ± 24	131 ± 26	136 ± 29
	Group V	125 ± 15	90 ± 8^c^	104 ± 14^c^
	Group S	120 ± 23	135 ± 26^d^	155 ± 29^c^
	Group SV	126 ± 10	106 ± 21^c^	117 ± 22
Mean arterial blood pressure (mm Hg)^a,b^				
	Group C	80 ± 12	80 ± 13	77 ± 13
	Group V	80 ± 9	97 ± 9^c^	100 ± 11^c^
	Group S	68 ± 9	67 ± 9	67 ± 7
	Group SV	74 ± 9	100 ± 22^c^	95 ± 20^c^
Cardiac index (mL/kg per minute)^a,b^				
	Group C	148 ± 31	147 ± 33	149 ± 34
	Group V	147 ± 27	96 ± 13^c^	118 ± 12^d^
	Group S	174 ± 22	166 ± 20	179 ± 14
	Group SV	168 ± 47	107 ± 13^c^	125 ± 27^c^
PAOP (mm Hg)^a,e^				
	Group C	6 ± 1	7 ± 1	6 ± 1
	Group V	6 ± 1	8 ± 1^d^	8 ± 2^c^
	Group S	8 ± 1	6 ± 1	6 ± 2
	Group SV	6 ± 1	6 ± 2	6 ± 2
Arterial pH				
	Group C	7.45 ± 0.03	7.45 ± 0.03	7.44 ± 0.04
	Group V	7.44 ± 0.02	7.44 ± 0.04	7.44 ± 0.05
	Group S	7.43 ± 0.02	7.44 ± 0.02	7.43 ± 0.02
	Group SV	7.43 ± 0.04	7.43 ± 0.04	7.43 ± 0.04
Arterial standard base excess (mmol/L)				
	Group C	3.9 ± 1.4	3.6 ± 1.5	3.3 ± 1.8
	Group V	2.4 ± 1.6	2.9 ± 1.9	2.9 ± 2.2
	Group S	3.1 ± 0.9	3.7 ± 0.5	3.2 ± 0.9
	Group SV	2.2 ± 1.2	3.0 ± 1.2	2.7 ± 1.5
Arterial lactate concentration (mmol/L)				
	Group C	0.99 ± 0.15	0.96 ± 0.12	0.95 ± 0.17
	Group V	0.98 ± 0.12	1.10 ± 0.24	1.11 ± 0.26^d^
	Group S	1.36 ± 0.48	1.11 ± 0.31^c^	1.10 ± 0.30^c^
	Group SV	1.46 ± 0.25	1.25 ± 0.17^c^	1.20 ± 0.16^c^
Arterial oxygen partial pressure (mm Hg)^f^				
	Group C	162 ± 19	156 ± 21	152 ± 25
	Group V	161 ± 16	143 ± 29^d^	141 ± 21
	Group S	163 ± 17	163 ± 16	161 ± 19
	Group SV	165 ± 10	157 ± 21	160 ± 13
Mixed venous oxygen saturation (percentage)^a,b,e^				
	Group C	66 ± 5	65 ± 6	66 ± 5
	Group V	64 ± 10	42 ± 10^c^	49 ± 11^c^
	Group S	59 ± 5	59 ± 5	61 ± 5
	Group SV	61 ± 6	53 ± 7^c^	58 ± 7
DO_2_I sys (mL/kg per minute)^a,b^				
	Group C	18 ± 2.6	18 ± 2.7	19 ± 2.8
	Group V	16 ± 2.8	9.4 ± 1.9^c^	11 ± 1.8^d^
	Group S	17 ± 2.1	18 ± 3.3	20 ± 2.8
	Group SV	19 ± 5.5	13 ± 2.2^c^	15 ± 3.6
VO_2_I sys (mL/kg per minute)				
	Group C	6.1 ± 0.8	6.0 ± 1.1	6.2 ± 1.1
	Group V	5.7 ± 0.7	5.6 ± 1.2	5.7 ± 1.1
	Group S	7.3 ± 1.3	7.3 ± 1.5	7.7 ± 0.5
	Group SV	7.5 ± 1.8	6.0 ± 0.9^d^	6.2 ± 1.1
Urinary output (mL/kg per hour)				
	Group C	2.1 ± 2.4	2.3 ± 2.4	1.8 ± 2.2
	Group V	1.6 ± 0.8	3.8 ± 2.2	5.4 ± 3.9^d^
	Group S	1.4 ± 0.9	1.9 ± 1.4	0.9 ± 0.5
	Group SV	1.0 ± 0.5	3.5 ± 3.2	2.6 ± 2.0

In groups V and SV, a continuous infusion of vasopressin (0.06 IU/kg per hour) was started at t = 300 minutes. Groups C and S received intravenous crystalloids only.

^a^*p *< 0.05 time-group interaction groups V versus C: effect of vasopressin in control animals.

^b^*p *< 0.01 time-group interaction groups SV versus S: effect of vasopressin in septic animals.

^c^*p *< 0.01 compared to t = 300 minutes.

^d^*p *< 0.05 compared to t = 300 minutes.

^e^*p *< 0.01 Mann-Whitney test (area under curve): effect of vasopressin in non-septic versus septic animals.

^f^*p *< 0.05 time-group interaction groups SV versus S: effect of vasopressin in septic animals.

DO_2_I sys: systemic oxygen delivery index. Group C: non-septic control group. Group S: septic control group. Group SV: septic test group treated with vasopressin. Group V: non-septic vasopressin control group. PAOP: pulmonary artery occlusion pressure. VO_2_I sys: systemic oxygen consumption index.

**Table 2:** Regional blood flow and oxygen delivery during infusion of vasopressin

Time		300 minutes	360 minutes	480 minutes
Celiac trunk (mL/kg per minute)^a,b^				
	Group C	11 ± 3	12 ± 2	12 ± 2
	Group V	17 ± 6	23 ± 5^c^	23 ± 5^c^
	Group S	19 ± 8	17 ± 5	16 ± 4
	Group SV	25 ± 17	31 ± 18^d^	30 ± 17^d^
Liver flow (mL/kg per minute)^a,b,e^				
	Group C	36 ± 3	35 ± 5	33 ± 4
	Group V	38 ± 9	31 ± 6^c^	33 ± 5^d^
	Group S	45 ± 8	37 ± 6^c^	35 ± 5^c^
	Group SV	43 ± 7	29 ± 6^c^	31 ± 6^d^
DO_2_I PV (mL/kg per minute)^a,b,e^				
	Group C	2.7 ± 0.4	2.6 ± 0.5	2.6 ± 0.4
	Group V	2.5 ± 0.6	1.2 ± 0.2^c^	1.4 ± 0.2^c^
	Group S	2.7 ± 0.5	2.2 ± 0.5^d^	2.1 ± 0.4^c^
	Group SV	3.1 ± 0.7	1.3 ± 0.6^c^	1.7 ± 0.4^c^
DO_2_I HA (mL/kg per minute)^a,b^				
	Group C	0.3 ± 0.3	0.4 ± 0.3	0.4 ± 0.3
	Group V	0.5 ± 0.2	0.8 ± 0.3	0.8 ± 0.2
	Group S	0.6 ± 0.3	0.6 ± 0.3	0.6 ± 0.3
	Group SV	0.6 ± 0.3	1.0 ± 0.7^c^	1.0 ± 0.7^c^
DO_2_I liver (mL/kg per minute)^a^				
	Group C	3.2 ± 0.4	3 ± 0.6	3.1 ± 0.5
	Group V	3.0 ± 0.7	2.0 ± 0.5^c^	2.2 ± 0.3^c^
	Group S	3.3 ± 0.6	2.8 ± 0.6	2.7 ± 0.6^d^
	Group SV	3.6 ± 0.8	2.3 ± 0.9^c^	2.6 ± 0.9^c^
Renal artery (mL/kg per minute)^a,b^				
	Group C	8.7 ± 1.3	9.3 ± 1.3	9.1 ± 1.5
	Group V	8.9 ± 2.2	7.6 ± 2.3^d^	8.9 ± 2.7
	Group S	8.1 ± 3.0	8.8 ± 3.5	9.0 ± 4.0
	Group SV	6.2 ± 2.3	5.4 ± 1.6	7 ± 2.4
DO_2_I kidney (mL/kg per minute)^a,e^				
	Group C	1.1 ± 0.2	1.1 ± 0.3	1.2 ± 0.3
	Group V	1.0 ± 0.2	0.7 ± 0.2^c^	0.9 ± 0.3
	Group S	0.7 ± 0.4	0.8 ± 0.5	0.9 ± 0.6^c^
	Group SV	0.7 ± 0.2	0.6 ± 0.2	0.8 ± 0.3^d^

A continuous infusion of vasopressin (0.06 IU/kg per hour) was started in groups V and SV at t = 300 minutes. Animals in groups C and S received intravenous saline only.

^a^*p *< 0.01 time-group interaction groups V versus C: effect of vasopressin in control animals.

^b^*p *< 0.01 time-group interaction groups SV versus S: effect of vasopressin in septic animals.

^c^*p *< 0.01 compared to t = 300 minutes.

^d^*p *< 0.05 compared to t = 300 minutes.

^e^*p *< 0.05 Mann-Whitney test (area under curve): effect of vasopressin in non-septic versus septic animals.

Celiac trunk: blood flow in the celiac trunk. DO_2_I HA: oxygen delivered by the hepatic artery. DO_2_I kidney: total oxygen delivered by the left renal artery. DO_2_I liver: total oxygen delivery to the liver. DO_2_I PV: oxygen delivered by the portal vein. Group C: non-septic control group. Group S: septic control group. Group SV: septic test group treated with vasopressin. Group V: non-septic vasopressin control group. Liver flow: total liver blood flow. Renal artery: blood flow in the left renal artery.

**Table 3:** Regional blood flow and oxygen delivery during infusion of vasopressin in non-septic animals

Time		300 minutes	360 minutes	480 minutes
Portal vein (mL/kg per minute)^a,b^				
	Group C	33 ± 3	32 ± 5	30 ± 4
	Group V	33 ± 8	23 ± 4^c^	25 ± 4^c^
Hepatic artery (mL/kg per minute)^a^				
	Group C	3.2 ± 1.7	3.4 ± 1.7	3.7 ± 1.7
	Group V	4.4 ± 1.6	8.2 ± 3.1^c^	8 ± 2.5^c^
MBF liver (percentage)				
	Group C	133 ± 53	135 ± 61	122 ± 57
	Group V	85 ± 22	75 ± 38	77 ± 44
MBF kidney (percentage)^a^				
	Group C	96 ± 14	94 ± 20	96 ± 20
	Group V	106 ± 15	86 ± 16^c^	105 ± 15
MBF pancreas (percentage)^a^				
	Group C	119 ± 14	108 ± 18^c^	99 ± 17^c^
	Group V	110 ± 30	74 ± 15^c^	76 ± 23^c^

A continuous infusion of vasopressin (0.06 IU/kg per hour) was started at t = 300 minutes in group V. Animals in group C received intravenous saline only.

^a^*p *< 0.01 time-group interaction groups V versus C: effect of vasopressin in control animals.

^b^*p *< 0.05 Mann-Whitney test (area under curve): effect of vasopressin in non-septic versus septic animals.

^c^*p *< 0.01 compared to t = 300 minutes.

Group C: non-septic control group. Group V: non-septic vasopressin control group. Hepatic artery: blood flow in the hepatic artery. MBF kidney: microcirculatory blood flow in the renal cortex. MBF pancreas: microcirculatory blood flow in the pancreas. MBF was measured by laser Doppler flowmetry and expressed as percentage of baseline. Portal vein: blood flow in the portal vein.

All animals in groups S and SV first developed signs of hypodynamic septic shock, with low MAP, low CI, and decreased microcirculatory blood flow, followed by signs of normo/hyperdynamic sepsis after fluid administration (Appendix 1). Fluid resuscitation increased CI. It restored blood flow in the portal vein, the celiac trunk, and the hepatic and renal arteries. Furthermore, it restored microcirculatory blood flow in the renal cortex. In contrast, fluid administration did not restore microcirculatory blood flow in the liver (down by 15% to 27%) or the pancreas (down by 27% to 32%).

Substantial effects of vasopressin on the systemic and regional circulation were observed within a few minutes after starting the vasopressin infusion (in groups V and SV). The peak effect on most systemic and regional parameters was measured between 30 and 60 minutes after starting vasopressin (Tables 1, 2, 3; Figures 1 and 2). Administration of vasopressin to septic animals (group SV) increased MAP and decreased CI and HR.

Administration of vasopressin resulted further in significant redistribution of splanchnic blood flow (Figure 1; Table 2): 60 minutes after the start of vasopressin infusion, blood flow in the portal vein had decreased by 58% in septic animals receiving vasopressin (group SV) but by 19% in septic controls (group S; *p *< 0.01). Blood flow in the celiac trunk increased by 20% in group SV and by 30% in group V but decreased by 15% in group S (Figure 1; Table 2). The hepatic artery blood flow remained virtually unchanged or increased in some animals (Figure 1). Thus, similar to portal flow, total liver blood flow decreased (*p *< 0.01) more in group SV (by 32%) than in group S (by 15%; Table 2). Microcirculatory blood flow in the liver remained unchanged in both septic groups. Administration of vasopressin in group SV decreased microcirculatory blood flow in the pancreas further to 36% ± 14% (*p *< 0.01) of baseline, whereas virtually no change occurred in group S.

Renal artery blood flow remained unchanged in septic controls (group S) as well as in septic animals receiving vasopressin (group SV; Table 2). In group SV, microcirculatory blood flow in the renal cortex decreased by 16% ± 20% (Figure 2; *p *< 0.01), but urine output increased (Table 1). Microcirculatory blood flow in group S remained unchanged. Systemic, regional, and microcirculatory flow parameters (Table 3) remained stable in control animals not receiving vasopressin (group C) and in vasopressin control animals (group V) during the first 300 minutes.

Administration of vasopressin to non-septic animals (group V) resulted in systemic, regional, and local changes similar to those seen in septic animals (Tables 1, 2, 3). However, the effects of vasopressin on some systemic (pulmonary artery occlusion pressure and mixed venous oxygen saturation) and regional (total liver blood flow, portal blood flow, portal oxygen delivery [DO_2_I PV], and renal oxygen delivery [DO_2_I kidney]) parameters appeared to be stronger in non-septic than in septic animals.

## Discussion

Two septic and another two non-septic groups were studied in a factorial design with the aim of comparing changes in systemic blood flow with changes in regional splanchnic blood flow and microcirculatory blood flow in multiple abdominal organs during administration of low-dose vasopressin in septic shock. Therefore, the results of the non-septic groups are presented for reference only and are not discussed in detail.

Administration of low-dose vasopressin in this porcine model of volume-resuscitated septic shock increased arterial blood pressure, decreased systemic blood flow and oxygen delivery, and resulted in a marked redistribution of blood flow in the splanchnic region. Portal venous flow decreased almost by half in the group receiving vasopressin. In contrast, hepatic arterial blood flow either remained unchanged or increased. This finding suggests different effects of vasopressin on the arterial versus the portal venous blood supply in the liver. In fact, it has been shown in non-septic rats that effects of vasopressin on the liver are heterogenous and more pronounced on the portal venous side than on the arterial side, due to receptor density, which favors the portal zone [29]. In non-septic low-flow states, liver blood flow is known to be regulated by the hepatic arterial buffer response, in which a decrease in portal flow leads to increased hepatic arterial blood flow due to vasodilatation, which is mediated locally by the accumulation of adenosine [30]. In the present study, hepatic arterial buffer response did not fully compensate for decreased portal flow, except perhaps in one animal out of eight (Figure 1). Our results in septic pigs are also in accordance with a study by Schiffer and colleagues [31] on endotoxic sheep showing that capacity of the hepatic arterial buffer response is diminished during endotoxemia [32].

Although total liver blood flow decreased during administration of vasopressin, average microcirculatory blood flow measured on the surface of the liver remained unchanged. This finding must be interpreted with caution. One question that has to be addressed is whether microcirculatory flow measured on the surface of the liver is representative of the entire organ. In rats, microcirculatory blood flow measured on the hepatic surface using LDF has been reported to reflect changes in total liver blood flow [33]. Similar findings were found in a porcine model [34]. However, the authors of the latter study also reported an increased sensitivity of LDF to changes in arterial blood flow [34].

Microcirculatory blood flow in the pancreas decreased markedly during the development of septic shock. Although intravenous fluids appeared to have effectively restored systemic and regional blood flows, microcirculatory flow in the pancreas remained approximately 30% below baseline after fluid administration. Administration of vasopressin further decreased pancreatic blood flow by approximately 50% despite the fact that blood flow in the supplying regional artery (celiac trunk) increased (Table 2). Why the hepatic artery was apparently getting a larger share of flow in the celiac trunk than the pancreas cannot be answered from the present data. It is possible that the V1 receptors (V1Rs) are more dense in the pancreatic vascular bed than in the hepatic artery or that, even if the hepatic arterial buffer response could not increase arterial hepatic flow enough to maintain liver blood flow unchanged, it may have limited the reduction in liver flow by reducing the resistance in the hepatic artery and thereby favoring distribution of flow in the celiac trunk to the liver.

Previous studies demonstrate that the pancreas is very vulnerable to deterioration of systemic and splanchnic blood flow caused by hemorrhage [19], sepsis [20,35], and administration of vasoconstrictors such as vasopressin under non-septic conditions [11,36,37]. We are not aware of any other study that has investigated the effects of vasopressin on the pancreas in septic shock. Hypoperfusion of the pancreas may be a clinically relevant problem; the pancreas has been suggested to be a source of toxic mediators after ischemia and reperfusion injury [38], and impaired pancreatic function has been found after prolonged hypoperfusion [18].

Blood flow in the renal artery decreased moderately after the vasopressin infusion began but recovered to baseline with time. Microcirculatory blood flow in the renal cortex also decreased but remained low. Despite decreased regional and microcirculatory blood flow in the kidney, urine output increased. Vasopressin produces vasoconstriction via the V1Rs, whereas osmoregulation, antidiuretic effects, and nitric-oxide-dependent vasodilatation are mediated via the V2 receptors (V2Rs) [39]. In the present study, we used the vasopressin analogue ornithin-8-vasopressin, which has effects very similar to those of arginine vasopressin but a slightly higher affinity for V1R. However, it can still bind to the V2R once V1Rs are saturated. There is experimental evidence that, in the kidney, vasopressin preferentially constricts efferent arterioles [40]. Thus, increased diuresis was related to increased filtration pressure rather than to renal blood flow. Increased diuresis during administration of vasopressin has also been reported in patients in septic shock [21] and with hepatorenal syndrome [41].

The aim of this study was to measure the effects of vasopressin on regional and microcirculatory blood flow in abdominal organs during septic shock. Severe, irreversible microcirculatory disturbances have been associated with poor outcome in patients with septic shock [42]. In patients dying from septic shock, these disturbances have been shown to persist even after correction of systemic variables [26,43]. Nevertheless, treatment of circulatory shock is mostly guided by systemic variables alone because direct measurements of regional and local splanchnic blood flow in patients are invasive, time-consuming, and require special skills and instruments that are not readily available at the bedside.

We intended to simulate clinical conditions in critically ill patients as closely as possible. The pig model appeared suitable because of the pig's anatomical and physiologic similarity to humans with respect to the cardiovascular system and the digestive tract [44,45]. Fecal peritonitis is a frequent cause of septic shock in humans, and clinical conditions in a critical care unit were imitated as closely as possible in the laboratory (sedation, mechanical ventilation, monitoring, and drug administration). Still, the results of this study are not based on human data, and that is the study's main limitation. Furthermore, due to the small number of animals per group, some biologically relevant effects may have been missed. The full factorial design used in this study, comprising three different control groups, was intended to minimize this risk. Another limitation of this study may be the fact that we measured only organ blood flow, but not metabolism. However, a recent study performed in our laboratory suggested that signs of anaerobic metabolism in tissues may be detected only relatively late and only when blood flow is substantially reduced (that is, by 60% or more) and that an even greater reduction of blood flow may be required in order to detect these signs in regional venous blood [46]. The vasopressin dose used in the present study (0.06 U/kg per hour, which is approximately 0.06 U/minute in a 70-kg human) was determined in pilot studies as the dose required to raise MAP by 20 to 25 mm Hg in septic pigs. This perhaps may be considered 'high' by some investigators since it is higher than the 0.04 U/minute proposed by several authors, including Malay and colleagues [14]. However, the dose used in the present study was lower than 0.10 U/minute, which Martikainen and colleagues [15] advised as the upper limit since higher doses caused reduction in systemic and splanchnic blood flow in endotoxemic animals. On the other hand, Klinzing and colleagues [47] administered vasopressin as a single vasopressor in septic patients in doses that were up to 50 times higher than those used in the present study (average, 27 U/minute).

Another matter that deserves mentioning here is the fact that most clinicians who use vasopressin in septic shock use it as a supplementary vasopressor to norepinephrine or in combination with dobutamine. However, in the present study, we used vasopressin alone because our purpose was to study the effects of vasopressin without possible interactions of other vasoactive agents.

## Conclusion

Administration of low-dose vasopressin in septic shock resulted in increased arterial blood pressure and decreased systemic blood flow. Splanchnic regional blood flow was substantially redistributed. It decreased markedly in the portal vein, but remained unchanged or increased in the hepatic artery, and increased in the celiac trunk. This resulted in significantly decreased total liver blood flow. Microcirculatory blood flow remained unchanged in the liver but decreased markedly in the pancreas. Initially, blood flow in the renal artery decreased, but it returned to baseline levels after 3 hours, whereas microcirculatory flow in the renal cortex remained decreased. This study also showed that increased urine output does not necessarily reflect increased renal blood flow. Considering these disturbances in blood flow and the fact that the safety of vasopressin in septic shock has not yet been demonstrated in humans, vasopressin should be used with great caution for treatment of hypotension in septic shock.

## Key messages

Administration of low-dose vasopressin in porcine septic shock resulted in:

• a fast and sustained increase in blood pressure and a marked decrease in systemic blood flow.

• significant redistribution of splanchnic regional and microcirculatory blood flow that could not be predicted or detected by systemic parameters.

• a decrease in portal blood flow of 58%. This decrease was compensated for, in part, by increased hepatic arterial blood flow. Nevertheless, total liver blood flow decreased by 32%.

• a decrease in microcirculatory blood flow in the pancreas by 36%. Hypoperfusion of the pancreas may be a relevant problem.

• increased urine output but decreased renal arterial and microcirculatory blood flow, indicating pressure diuresis.

## Abbreviations

ANOVA = analysis of variance; CaO_2 _= arterial oxygen content; CI = cardiac index; CO = cardiac output; CVP = central venous pressure; DO_2 _= oxygen delivery; DO_2_I = oxygen delivery index; FiO_2 _= fraction of inspired oxygen; Hb = hemoglobin concentration; HR = heart rate; LDF = laser Doppler flowmetry; MAP = mean arterial blood pressure; PAP = pulmonary artery pressure; PCWP = pulmonary capillary wedge pressure; PEEP = positive end-expiratory pressure; SVR = systemic vascular resistance; V1R = V1 receptor; V2R = V2 receptor.

## Competing interests

The authors declare that they have no competing interests.

## Authors' contributions

VK participated in the experimental design, animal preparation and performance and supervision of experimental work, preliminary analysis of the data, and writing of the manuscript. LH participated in the experimental design, animal preparation and performance and supervision of experimental work, and preliminary analysis of the data and helped to draft the manuscript. SJ provided assistance and consulting during the experimental design, provided statistical analysis, and helped to draft the manuscript. JT provided assistance and consulting of the experimental design and helped to finalize the manuscript, in particular the Discussion section. GS provided assistance and consulting of the experimental design and helped to finalize the manuscript, in particular the Discussion section, and provided supervision and overview of the entire project. All authors read and approved the final manuscript.
